# Ternary Synergistic Electrolyte Enabling Stable Li-Ion Battery Operation Across −40 °C to 60 °C

**DOI:** 10.3390/ma18204803

**Published:** 2025-10-21

**Authors:** Yali Zhao, Yutao Liu, Jingju Liu, Daofa Ying, Xuanlin Gong, Linjin Xie, Xiaohan Guo, Caiyun Yao, Baohui Chen, Chuanping Wu

**Affiliations:** 1 State Key Laboratory of Disaster Prevention and Reduction for Power Grid Transmission and Distribution Equipment, State Grid Hunan Electric Company Limited Disaster Prevention and Reduction Center, Changsha 410007, China; ytliu_whu@126.com (Y.L.); liujingj132@163.com (J.L.); 17364041396@163.com (D.Y.); bymountains@gmail.com (B.C.); jandom@126.com (C.W.); 2 Hunan Disaster Prevention Technology Co., Ltd., Changsha 410100, China; 119031910101@sjtu.edu.cn (X.G.); 18684788989@163.com (L.X.); guox46057@gmail.com (X.G.); ccyao929@163.com (C.Y.)

**Keywords:** all-climate electrolyte, lithium-ion battery, solid electrolyte interphase, lithium difluorophosphate, methyl acetate

## Abstract

The operational failure of lithium-ion batteries under extreme temperatures (−40~60 °C) stems primarily from electrolyte limitations. While prior efforts improved either low-temperature or high-temperature performance independently, holistic electrolyte design with practical validation remains elusive. Herein, we develop an all-climate electrolyte (ACE) through synergistic coordination of solvent, Li salt, and additive, achieving low viscosity (<10 mPa·s at −40 °C) and high ionic conductivity (7.0 mS cm^−1^ at −40 °C). Raman and NMR spectra reveal MA and EC co-occupying Li^+^ solvation sheath while EMC acts as a diluent, enabling rapid ion transport. Consequently, LiFePO_4_ (LFP)|graphite (Gr) cell delivers unprecedented cyclability: zero capacity decay over 500 cycles at 0 °C, stable operation across −40~60 °C, and 94.1% retention after 100 cycles at 45 °C in Ah-level pouch cells. XPS and SEM analysis demonstrate lithium difluorophosphate (LiDFP) and lithium bis(fluorosulfonyl)imide (LiFSI) collaboratively remodel SEI/CEI interphases, enriching them with LiF, Li_3_PO_4_, and Li_2_SO_4_. This inorganic-dominant architecture enhances interfacial Li^+^ kinetics and all-climate stability compared to the baseline electrolyte. Our tripartite electrolyte strategy provides a material-agnostic solution for all-climate energy storage.

## 1. Introduction

Lithium-ion batteries (LIBs) represent the cornerstone of modern energy storage systems, powering applications from portable electronics to electric vehicles. However, their operational reliability across extreme temperatures (−40~60 °C) remains a critical challenge, largely constrained by the physicochemical limitations of conventional electrolytes [1,2,3]. The liquid–solid phase transitions, viscosity surge at low temperatures, and interfacial instability at high temperatures collectively deteriorate ion transport and electrode kinetics, impeding practical deployment in all-climate scenarios.

Significant efforts have been devoted to developing wide-temperature electrolytes. For low-temperature performance, carboxylate esters were introduced to suppress crystallization, yet their poor oxidative stability and instability raise concerns at elevated temperatures [4,5,6]. Conversely, high-temperature stability has been addressed via thermally stable lithium salts [7,8], high-boiling-point solvents [9,10], or film-forming additives [11,12], often at the expense of low-temperature ionic conductivity. For example, Wang et al. [13] reported that 1 M LiTFSI in CPME/FEC (7/3) could operate at temperatures as low as −40 °C, delivering a capacity of 0.1 Ah. Similarly, Yang et al. [14] demonstrated that 1 M LiTFSI in ETFA/FEC (7/3) functioned down to −70 °C, achieving a specific capacity of 43 mAh g^−1^. However, these studies primarily focus on low-temperature performance, offering little insight into high-temperature stability. In contrast, a Li secondary battery employing 1 M LiFSI in THP electrolyte [15] exhibited a wide operating temperature range from −20 °C to 60 °C, with 2.25 Ah retained at −20 °C and 96.5% capacity retention after 300 cycles at 60 °C in Ah-level pouch cells. Li et al. [16] further demonstrated that 1.5 M LiFSI in MP/FEC (9/1) electrolyte not only delivered 78 mAh g^−1^ at −30 °C but also maintained a high capacity of 115 mAh g^−1^ at 80 °C. Nevertheless, a systematic design framework that concurrently tailors salt chemistry, solvent composition, and additive functionality is still missing, limiting the rational development of high-performance electrolytes. Moreover, validation in practical Li-ion batteries remains limited, posing a barrier to translating material innovations into real-world applications.

Herein, we propose an all-climate electrolyte (ACE) engineered through the ternary synergistic effect of salt, solvent, and additive. The ACE integrates methyl acetate (MA) for an extended liquidus range, lithium bis(fluorosulfonyl)imide (LiFSI) for enhanced ionic dissociation, and lithium difluorophosphate (LiDFP) as an interfacial modifier, achieving ultralow viscosity (<10 mPa·s at −40 °C) and high ionic conductivity (~7 mS cm^−1^ at −40 °C). Combined Raman and NMR analyses reveal a unique solvation structure where MA and EC coordinate Li^+^ while EMC acts as a diluent, thereby optimizing bulk transport properties. Crucially, LFP|Gr cells with ACE exhibit exceptional cycling stability (zero capacity decay over 500 cycles at 0 °C, stable operation across −40~60 °C and 94.1% retention after 100 cycles at 45 °C in Ah-level pouch cells). XPS and SEM confirm that LiDFP and LiFSI collaboratively remodel the SEI and CEI, enriching them with Li_3_PO_4_, LiF, and Li_2_SO_4_. This inorganic-dominant interphase architecture significantly enhances interfacial Li^+^ flux and all-climate stability, ultimately unlocking the all-climate performance.

## 2. Experimental Sections

### 2.1. Materials

Lithium bis(fluorosulfonyl)imide (LiFSI, 99.8%), lithium difluorophosphate (LiDFP, 99.8%), methyl acetate (MA, 99.8%) were purchased from DoDo chem (Suzhou, China) and used as received. Ethyl Acetate (EA, 99.0%), methyl propionate (MP, 99.0%), ethyl propionate (EP, 99.0%), ethyl butyrate (EB, 99.0%), ethyl difluoroacetate (DFEA, 99.0%), ethyl trifluoroacetate (TFEA, 99.0%) were purchased from Sigma-Aldrich (St. Louis, MO, USA). All the solvents were dehydrated with 4A molecular sieve before use. 1 M LiPF_6_ in EC:EMC:DMC (*v*:*v*:*v* = 1:1:1) was purchased from Zhangjiagang Guotai Huarong New Chemical Materials Co., Ltd. (Zhangjiagang, China), and used as the baseline electrolyte.

### 2.2. Preparation of Electrolyte and Electrode

All the electrolytes used in experiments were prepared by adding a certain amount of Li salt to the mixed solvents and stirring at room temperature for 24 h before use. The all-climate electrolyte (ACE) was 1 M LiFSI in MA:EC:EMC (*v*:*v*:*v* = 1:1:1) + 0.5 wt.% LiDFP. All the preparation was conducted in an Ar-filled glovebox (H_2_O, O_2_ < 0.1 ppm).

Commercial LFP (LF0608) and Gr (SM0205) electrodes were purchased from Canrd company (Dongguan, China); the active material ratios were 91.5% and 95.7%, respectively. The areal capacity was ~1.58 mAh cm^−2^ (~10.5 mg cm^−2^) for LFP, and ~1.89 mAh cm^−2^ (~5.55 mg cm^−2^). All the electrodes were vacuum dried for 12 h before use. Areal capacity (mAh cm^−2^) denotes the capacity per unit electrode area, obtained by multiplying the specific capacity (mAh g^−1^) by the areal loading of active material (mg cm^−2^). It provides a practical measure of the electrode’s energy storage capability under realistic mass loading conditions. Mathematically, the areal capacity Careal can be expressed as

$$C_{areal}=C_{spec}{\;\times m}_{loading}$$

where *C_spec_* is the specific capacity of the electrode (mAh g^−1^), and *m_loading_* is the active material mass loading (g cm^−2^).

### 2.3. Preparation of LFP|Gr Pouch Cell

The LFP cathode comprises LFP, SP C60, CNTs, and PVDF in a mass ratio of 96:1:1:2. The slurry was coated on both sides of carbon-coated Al foils, corresponding to a mass loading of LFP of 20 mg cm^−2^ for each side, providing a capacity of ~3 mAh cm^−2^, calculated based on the specific capacity of LFP (150 mAh g^−1^). The Gr anode was formulated with Gr, SP, CMC, PAALi, and SBR in a mass ratio of 96.1:1:0.7:1.2:1. The slurry was coated on both sides of Cu foils, the mass loading of Gr being ~9.7 mg cm^−2^, corresponding to a capacity of ~3.3 mAh cm^−2^, calculated based on the specific capacity of Gr (340 mAh g^−1^). The negative/positive (N/P) ratio was set as ~1.10. The sizes of the cathode and anode are 39 mm * 79 mm and 39 mm * 80.5 mm, respectively. Typically, 10 pieces of LFP electrodes and 11 pieces of Gr electrodes were employed to obtain a ~2 Ah LFP|Gr pouch cell. Celgard 2325 was used as the separator, and the amount of electrolyte was 4 g Ah^−1^. The dry LFP|Gr pouch cells were vacuum dried at 90 °C for 24 h before injection of the electrolyte in an Ar-filled glove box. During the formation process, all the pouch cells were charged at 0.1C for 10 min and 0.16C for 110 min with an external pressure of 700 kPa at 45 °C. The air bag was then removed, and the pouch cells were sealed under vacuum. After that, the pouch cells were cycled between 2.5~3.65 V at 0.1C for fully activation. All the pouch cells were then cycled between 2.5~3.65 V at 0.5C for the long-term stability evaluation at a Landt battery testing system (Wuhan LAND Electronic Co., Ltd., Wuhan, China).

### 2.4. Electrochemical Measurement

Cycling tests of CR2032 coin cells were conducted on the LAND electrochemical testing system (LANHE, Dongguan, China). The LFP|Li and LFP|Gr cells were cycled between 3.65~2.5 V, and three formation cycles at 0.1C were conducted for each cell. The formation cycle refers to several controlled charge–discharge cycles performed after cell assembly to stabilize the electrode/electrolyte interface, the solid electrolyte interphase (SEI) layer, pore wetting, and electrochemical contact within the electrode structure. The activation stage is typically conducted at a low current for three cycles within the voltage window of 2.5–3.65 V at different temperatures, in order to minimize irreversible side reactions and to form a stable interfacial structure.

The electrochemical stability of the electrolyte was determined by linear sweep voltammetry (LSV) test, where Al foil (positive scan) or Cu foil (negative scan) was used as the working electrode, and Li foil was used as the counter electrode and reference electrode. The potential range was set from open-circuit potential (OCP) to 5.0 V or −0.2 V (vs. Li^+^/Li), and the scanning rate was 0.1 mV s^−1^.

### 2.5. Characterization

Morphology was observed on a scanning electron microscope (SEM, ZEISS Merlin Compact (Carl Zeiss Microscopy GmbH, Fremont, CA, USA), operating at 3 kV accelerating voltage, 60 pA beam current, and a working distance of ~8 mm). Samples were mounted on 12.5 mm aluminum stubs using conductive carbon tape (SPI Supplies, West Chester, PA, USA). Electrodes acquired from cycled cells were thoroughly washed with dimethyl carbonate (DMC) and dried in an Ar-filled glovebox prior to transfer for SEM analysis. Surface chemical species on the anode were determined by X-ray photoelectron spectroscopy (XPS, Thermo Scientific ESCALAB 250Xi, Thermo Fisher Scientific, Waltham, MA, USA) with a monochromatic Al Kα X-ray source (1486.6 eV, 650 μm spot size) under ultra-high vacuum (<1 × 10^−9^ mbar). Data interpretation for both SEM and XPS was carried out using the built-in ZEN (Zeiss, Oberkochen, Germany) and Thermo Avantage 5.52 software, respectively. Raman spectra were collected with a Renishaw InVia Raman spectrometer (Renishaw plc, Gloucestershire, UK) (532 nm laser excitation, 1 mW laser power, spectral range 400–4000 cm^−1^), and baseline correction was performed using the LabSpec6 software. The ^1^H NMR spectra were obtained on a Bruker AVANCE 400 spectrometer (Bruker Corp., Billerica, MA, USA), with ~10 mg of sample dissolved in 0.6 mL of deuterated dimethyl sulfoxide (DMSO-d_6_) containing tetramethylsilane (TMS) as the internal reference. Spectra were acquired and processed with MestReNova 11 software. The ionic conductivity and viscosity of the electrolytes were tested with a DDBJ-350 conductivity meter (Leici, Shanghai, China) and an MCR 302e rheometer (Anton Paar, Graz, Austria) at various temperatures. All electrolyte samples were sealed in glass capillaries with vacuum silicone grease in an Ar-filled glovebox to prevent moisture interference.

## 3. Results and Discussion

### 3.1. Electrolyte Design and Physicochemical Properties

**Figure 1:**
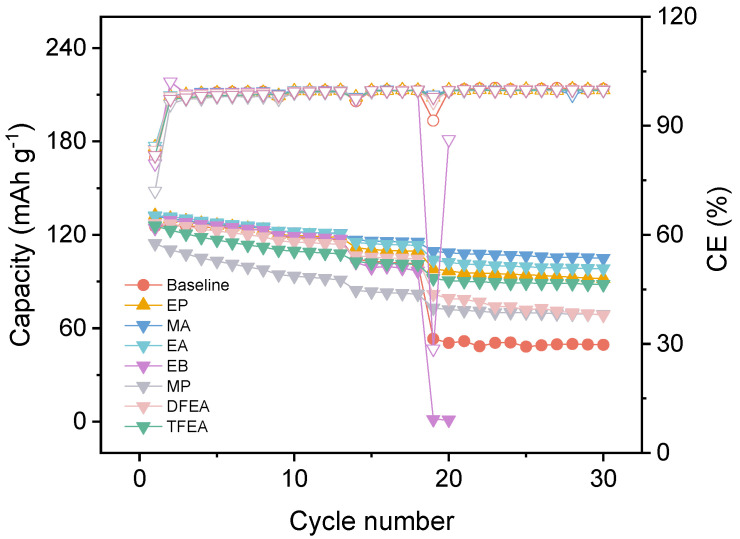
Electrochemical performance of LFP|Gr cells with various electrolytes (the solid triangles correspond to the discharge capacity on the left *y*-axis, while the empty triangles correspond to the Coulombic Efficiency on the right *y*-axis.).

To broaden the liquidus range of the electrolyte while preserving film-forming capability, we maintained the ethylene carbonate (EC) content unchanged and systematically evaluated various carboxylate ester solvents as replacements for dimethyl carbonate (DMC). Concurrently, to enhance the high-temperature stability, the thermally superior LiFSI was employed to replace the conventional LiPF_6_ salt. Additionally, to compensate for the insufficient film-forming ability of EC at elevated temperatures, lithium difluorophosphate (LiDFP) additive was also introduced at a concentration of 0.5 wt.%. The ionic conductivities of the resulting electrolytes were summarized in Table 1, which clearly indicated significant variations upon the incorporation of different carboxylate esters, with methyl acetate (MA) yielding the most substantial enhancement. Furthermore, the impact of these carboxylate esters on electrochemical performance was assessed. As demonstrated in Figure 1, the electrolyte incorporating MA maintained excellent compatibility within LFP|Gr cells and contributed to improved rate capability compared to other electrolytes. Consequently, MA was selected as the optimal co-solvent for subsequent investigations, and this optimized electrolyte comprising MA, LiFSI, and LiDFP is hereafter designated as the all-climate electrolyte (ACE).

**Figure 2:**
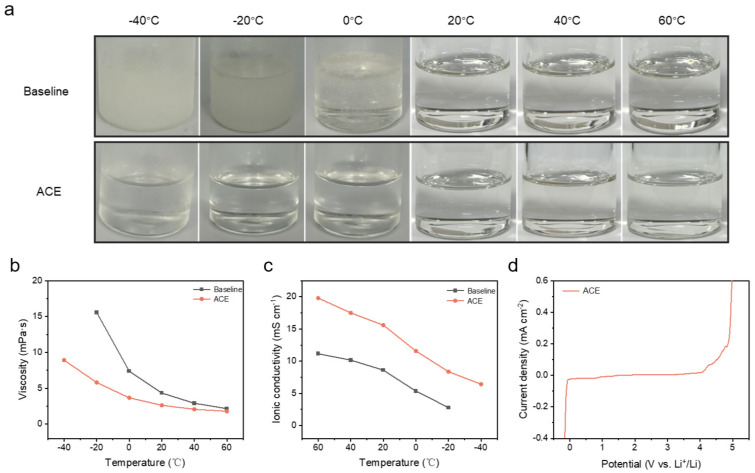
(**a**) Optical images of electrolyte from −40~60 °C. (**b**) Viscosity, (**c**) ionic conductivity, and (**d**) electrochemical window of different electrolytes.

To investigate the properties of the ACE across different temperatures, we first visually assessed the physical state of the electrolytes from −40~60 °C. As demonstrated in Figure 2a, the baseline electrolyte began to crystallize at −20 °C, attributable to the high melting point of EC. Upon further cooling to −40 °C, the baseline electrolyte solidified completely. In striking contrast, the ACE remained in a liquid state even at −40 °C, critically confirming its significantly broadened liquidus range. To quantitatively compare the transport properties, we further measured the viscosity and ionic conductivity of the ACE and baseline electrolytes across the temperature range, as presented in Figure 2b,c. The viscosity of the baseline electrolyte exhibited a dramatic increase with decreasing temperature, exceeding 15 mPa·s at −20 °C, which renders it practically unsuitable for low-temperature operation. Conversely, the ACE maintained a substantially lower viscosity, remaining below 10 mPa·s even at the extreme temperature of −40 °C. Furthermore, the ACE demonstrated superior ionic conductivity compared to the baseline electrolyte across the entire tested temperature spectrum. Remarkably, the ACE retained a conductivity of ~7 mS cm^−1^ at −40 °C, which is comparable to that of the baseline electrolyte measured at 10 °C. This exceptional enhancement in low-temperature transport properties should be primarily attributed to the combined effects of the low viscosity and low melting point of MA, as well as the favorable dissociation characteristics of LiFSI. Finally, the electrochemical stability window was evaluated via linear sweep voltammetry (LSV). As shown in Figure 2d, the ACE displayed excellent electrochemical stability within the voltage range of 0 V to 4.2 V, fully meeting the operational requirements of LFP|Gr batteries.

### 3.2. Solvation Environment Characterization

**Figure 3:**
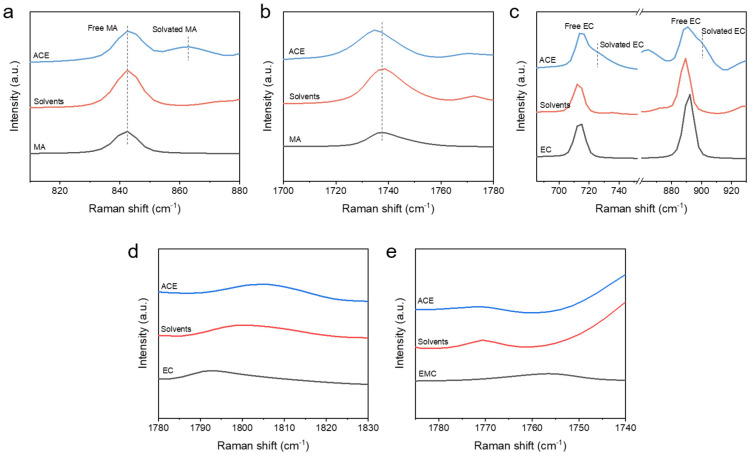
Raman spectra of pure single solvents, mixed solvents, and ACE. (**a**,**b**) MA characteristic range. (**c**) EC characteristic range. (**d**,**e**) EMC characteristic range.

The solvation structure of an electrolyte plays a decisive role in determining its physicochemical properties [17,18]. Therefore, gaining a deep understanding of the solvation characteristics within the ACE was essential. To achieve this target, Raman spectroscopy was performed. Given that MA possesses a higher donor number (DN) than EC, it was theoretically expected to participate in the Li^+^ solvation sheath [19,20]. This hypothesis was confirmed by the Raman result. As shown in Figure 3a,b, the pure MA spectrum exhibited characteristic peaks at ~843 cm^−1^ and ~1737 cm^−1^, corresponding to free MA. Upon the introduction of LiFSI, a new peak emerged at ~865 cm^−1^, assigned to coordinated MA. Concurrently, the peak at ~1737 cm^−1^ underwent a noticeable redshift, which was also derived from the interaction between MA and Li^+^. Similar spectral changes were observed for EC (Figure 3c). New peaks appeared at ~725 cm^−1^ and ~900 cm^−1^ corresponding to coordinated EC upon LiFSI addition. These results collectively demonstrated that both MA and EC entered the Li^+^ solvation shell in the ACE. As a linear carbonate, EMC exhibited a significantly weaker coordination ability towards Li^+^. This was evidenced by the minor changes in Raman spectra upon LiFSI addition (Figure 3d,e).

**Figure 4:**
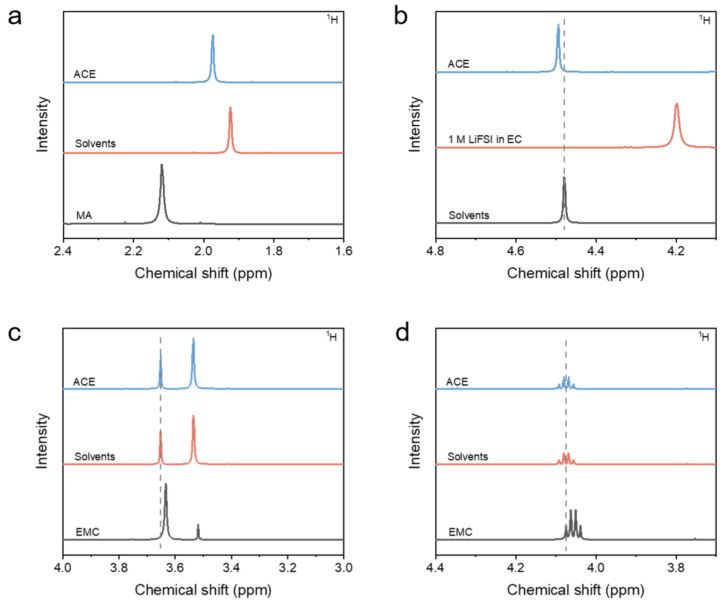
^1^H NMR spectra of ATE. (**a**) MA characteristic signals. (**b**) EC characteristic signals. (**c**,**d**) EMC characteristic signals.

NMR spectroscopy provided further insights into the weak interactions among solvents within the ACE. As shown in Figure 4a, the ^1^H NMR signal of MA shifted upfield upon mixing with the other solvents (EC and EMC), suggesting potential electron-donating effects from EC and/or EMC towards MA. Significantly, upon the addition of LiFSI, the ^1^H NMR signal of MA shifted downfield, providing further evidence for its pronounced coordination with Li^+^. Due to the solid state of EC at room temperature, 1 M LiFSI EC electrolyte was prepared for comparative analysis. As depicted in Figure 4b, the ^1^H NMR signal of EC in the 1 M LiFSI EC electrolyte shifted upfield compared to its position in the mixed solvent (EC:EMC:MA) without LiFSI, despite the influence of Li^+^ coordination. This observation indicated that EC experienced a strong electron-withdrawing effect during the solvent mixing process, consistent with the conclusion drawn from Figure 4a. Nevertheless, the ^1^H NMR signal of EC in the ACE shifted downfield compared to its position in the mixed solvents, confirming that EC also participated in Li^+^ coordination. Comparing the extent of the downfield shifts observed for MA and EC, the coordination strength between MA and Li^+^ appeared stronger than that between EC and Li^+^, which aligns with the higher DN of MA. Finally, the changes in the ^1^H NMR signals of EMC were presented in Figure 4c,d. The signal of pure EMC also exhibited some shift compared to its position in the mixed solvents, indicating interactions between EMC and MA/EC, although the strength of these interactions was markedly weaker than those observed for MA and EC. Upon the introduction of LiFSI, the EMC signal showed almost negligible shift, demonstrating the weakest coordination ability towards Li^+^ among the three solvents.

### 3.3. Electrochemical Performance

**Figure 5:**
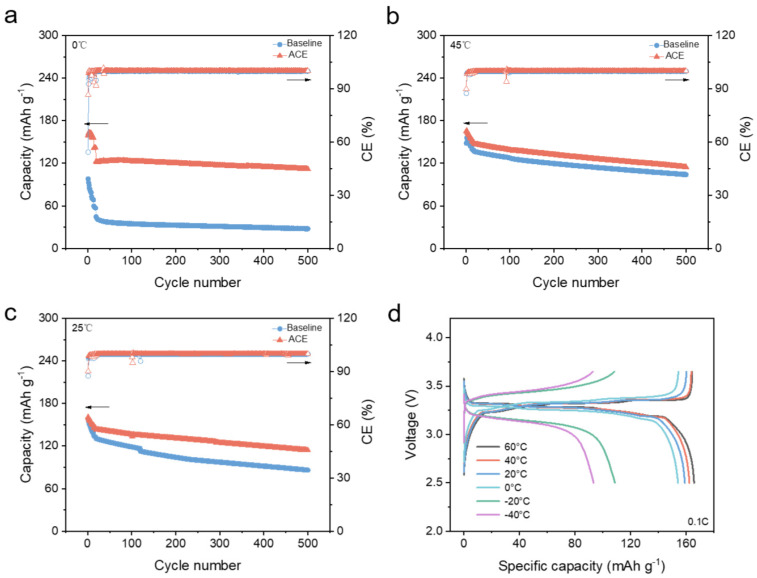
Electrochemical performance of coin cells with different electrolytes: (**a**) 0 °C, (**b**) 45 °C, and (**c**) 25 °C (the solid triangles correspond to the discharge capacity on the left *y*-axis, while the empty triangles correspond to the Coulombic Efficiency on the right *y*-axis.). (**d**) Charge/discharge profile of ACE-based LFP|Gr cell at various temperatures.

To confirm the long-term performance of the ACE in LFP|Gr batteries, evaluations were conducted in LFP|Gr coin cells. As shown in Figure 5a, the LFP|Gr coin cell employing the baseline electrolyte exhibited rapid capacity fading at 0 °C, delivering only ~39 mAh g^−1^. This observation aligns with the limited liquidus range identified earlier (Figure 2). By contrast, the ACE-based LFP|Gr cell maintained a significantly higher capacity of ~122 mAh g^−1^ at 0 °C, representing a threefold improvement over the baseline electrolyte. Furthermore, the ACE-based cell showed no noticeable degradation even after 500 cycles, demonstrating its exceptional stability at low temperature. Given the high content of MA, the stability of ACE at elevated temperatures traditionally raises concerns. Therefore, we specifically evaluated high-temperature performance. Remarkably, the ACE significantly outperformed the baseline electrolyte even at 45 °C (Figure 5b), retaining a capacity exceeding 115 mAh g^−1^ after 500 cycles. This superior high-temperature stability can be attributed to the synergistic effects of LiFSI and LiDFP, which effectively stabilize the electrolyte-electrode interphases [21,22]. Finally, the cycling performance at room temperature was also investigated. Consistent with the trends observed at extreme temperatures, the ACE continued to significantly outperform the baseline electrolyte under ambient conditions (Figure 5c). Representative charge/discharge voltage profiles of the ACE-based LFP|Gr cell across the operational temperature range (−40~60 °C) are presented in Figure 5d. The curves consistently maintained their characteristic shapes and demonstrated moderate polarization throughout the entire temperature window, highlighting the stable kinetics enabled by the ACE.

**Figure 6:**
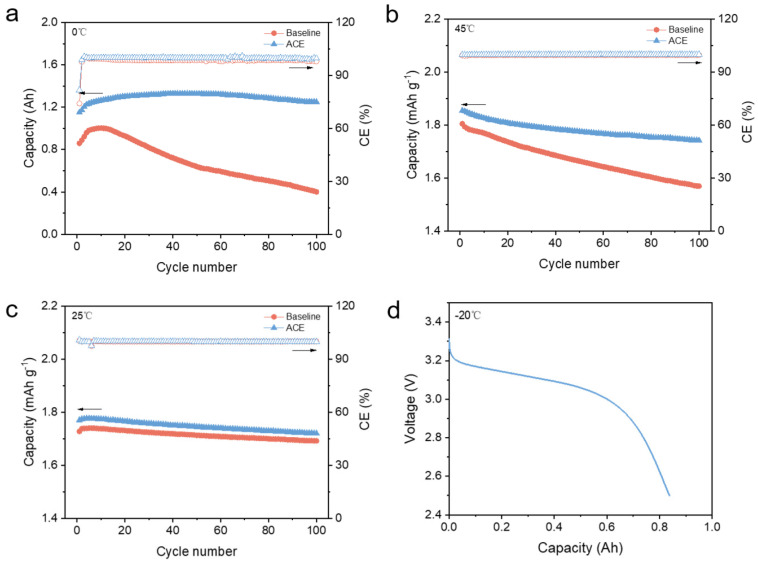
Electrochemical performance of Ah-level LFP|Gr pouch cells with different electrolytes at (**a**) 0 °C, (**b**) 45 °C, and (**c**) 25 °C (the solid triangles correspond to the discharge capacity on the left *y*-axis, while the empty triangles correspond to the Coulombic Efficiency on the right *y*-axis.). (**d**) discharge curve of the ACE-based LFP|Gr pouch cell at −20 °C.

To more closely replicate the operating condition of lithium-ion batteries in practical applications, validation was further performed using Ah-level pouch cells. As shown in Figure 6a, the pouch cell employing the baseline electrolyte exhibited significant capacity fade within only 100 cycles at room temperature. This rapid degradation may be associated with lithium plating issues of Gr anode induced by poor kinetics at low temperatures [23,24]. By contrast, the ACE maintained excellent stability, showing almost negligible capacity loss after 100 cycles. Furthermore, capacity fade in LFP|Gr pouch cells at 45 °C was accelerated compared to that in coin cells. The baseline electrolyte delivered a capacity retention of merely 84.9% after 100 cycles. Benefiting from the synergistic effects of LiFSI and LiDFP, the ACE achieved a significantly higher capacity retention of 93.92% after 100 cycles at 45 °C. Additionally, the room-temperature cycling performance of both electrolytes was examined. Although both electrolytes exhibited good stability at room temperature, the ACE-based LFP|Gr pouch cell consistently delivered higher specific capacities. This enhanced capacity should be attributed to ACE’s ability to reduce cell polarization. Finally, discharge capacity retention tests were conducted at an extreme low temperature of −20 °C. Remarkably, compared with previously reported wide-temperature lithium electrolytes (Table 2), the ACE exhibits higher ionic conductivity and overall superior performance. This work not only achieves a significant advancement in wide-temperature energy storage but also provides valuable insights into the design of electrolytes for wide-temperature rechargeable batteries. The ACE demonstrates commendable performance even under harsh conditions, further confirming its all-climate applicability.

### 3.4. Electrode/Electrolyte Interphase

**Figure 7:**
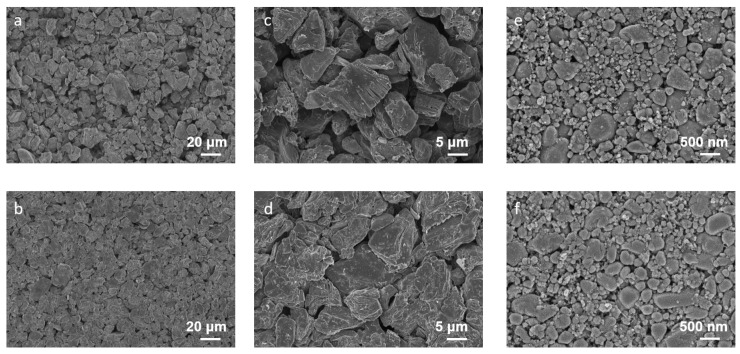
SEM images of Gr anode and LFP cathode cycled at 0 °C. (**a**,**c**) Gr anode cycled in baseline electrolyte. (**b**,**d**) Gr anode cycled in ACE. (**e**) LFP cathode cycled in baseline electrolyte. (**f**) LFP cathode cycled in ACE.

The outstanding electrochemical performance of the ACE is intimately linked to the unique characteristics of the electrode/electrolyte interphases. Considering that the performance improvement of ACE over the baseline electrolyte was most pronounced at low temperature, we specifically investigated the interphases formed after cycling at 0 °C. As revealed in Figure 7, distinct morphological differences were observed on the Gr anodes cycled at 0 °C. The Gr anode cycled in the baseline electrolyte exhibited a significantly more loosely packed structure, which likely resulted from uneven electrode expansion during cycling. Furthermore, pronounced delamination was evident, indicating that 0 °C cycling severely damaged the layered graphite structure. In stark contrast, the Gr anode cycled in the ACE remarkably retained its structural integrity. Compared to the dramatic changes on the Gr anode, the LFP cathodes showed much subtler morphological variations after cycling at 0 °C. The only discernible difference was that the LFP cathode surface cycled in ACE appeared rougher and was covered by a distinct layer of particles. This observed surface modification presumably correlates with compositional changes within the CEI formed in ACE.

**Figure 8:**
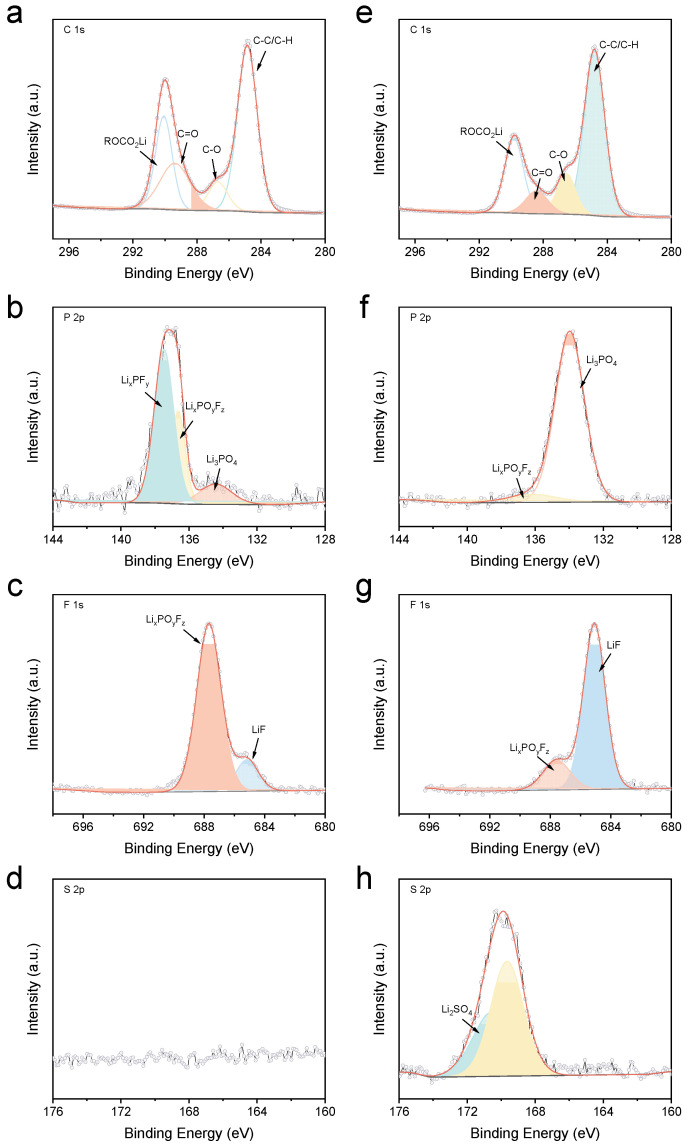
XPS profile of SEI formed in different electrolytes: (**a**–**d**) baseline electrolyte and (**e**–**h**) ACE.

XPS analysis provided further insights into the composition of the electrode/electrolyte interphases. As shown in Figure 8, while the overall C 1s spectra for the ACE-derived SEI appeared similar to that formed in the baseline electrolyte, significant differences were observed in the P 2p spectra. Specifically, the intensity corresponding to phosphorus oxyfluoride compounds (Li_x_PO_y_F_z_) substantially decreased in the ACE-derived SEI. Concurrently, a new prominent peak assigned to Li_3_PO_4_ emerged. These compositional changes were likely attributed to the involvement of LiDFP in forming the SEI, which was further corroborated by the F 1s spectra. The signal intensity for Li_x_PO_y_F_z_ notably diminished, while a substantial increase in the LiF signal intensity was observed. Additionally, distinct signals were clearly detectable in the S 2p spectra, indicating that LiFSI also actively participated in SEI formation, rather than merely enhancing the bulk ionic conductivity of the electrolyte.

**Figure 9:**
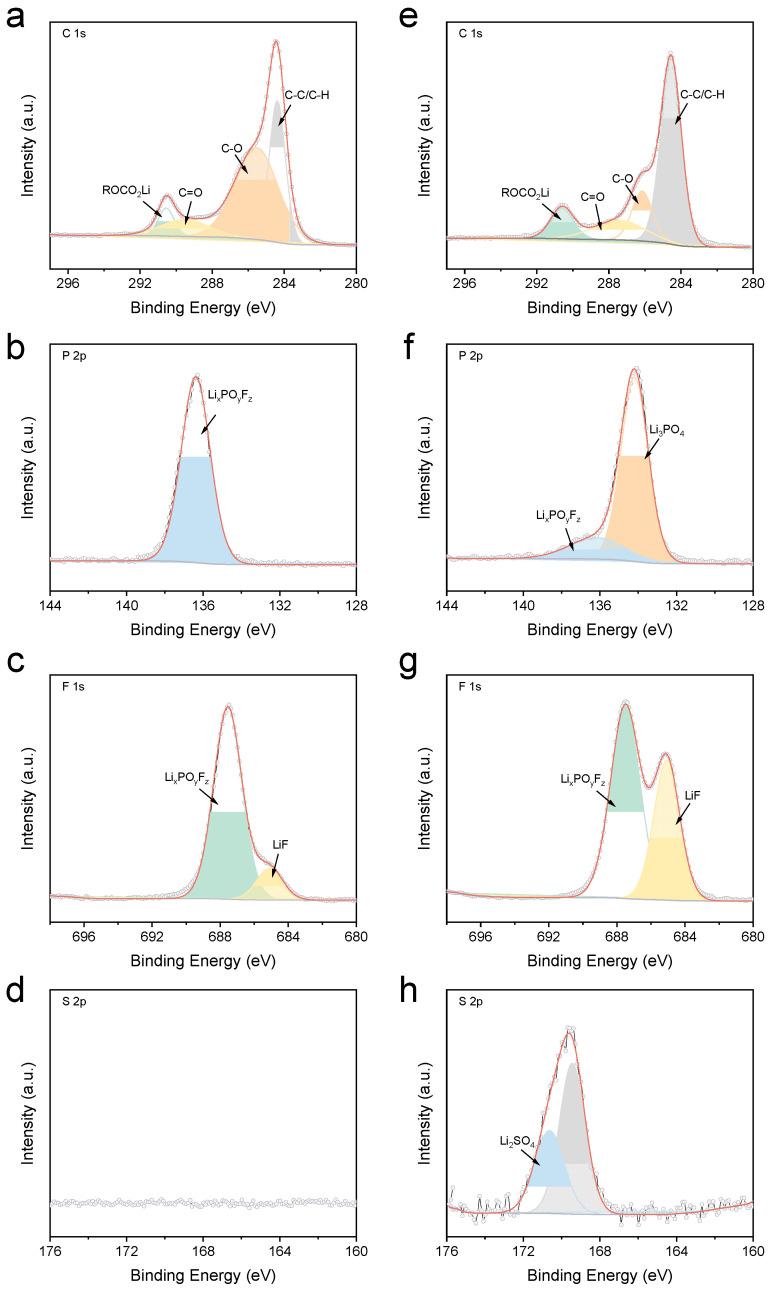
XPS profile of CEI formed in different electrolytes: (**a**–**d**) baseline electrolyte and (**e**–**h**) ACE.

Similarly, the composition of the CEI was comparatively analyzed, with the results presented in Figure 9. The C 1s spectrum derived from the ACE remained similar to that of the baseline electrolyte, indicating negligible changes in the organic constituents. Nevertheless, significant differences were observed in the P 2p and F 1s spectra. Consistent with the SEI findings, the signals corresponding to Li_x_PO_y_F_z_ markedly weakened, and a new peak was assigned to the emerged Li_3_PO_4_. Additionally, the LiF signal intensity also notably increased. These changes clearly demonstrated that LiDFP also participated in CEI formation. Finally, the distinct signals detected in the S 2p spectra confirmed that LiFSI similarly contributed to the formation of the CEI.

In summary, SEM and XPS results conclusively establish that the ACE effectively modified the composition of both the SEI and CEI, which originated predominantly from the LiDFP additive and the LiFSI salt. The resulting interphases were enriched with inorganic components like Li_3_PO_4_, LiF, and Li_2_SO_4_, which collectively enhance interfacial Li^+^ conduction and significantly improve interfacial stability. This optimized interphase underpins the exceptional all-climate electrochemical performance of the ACE.

## 4. Conclusions

In summary, this work demonstrates that the synergistic effect of the MA solvent, LiFSI salt, and LiDFP additive in an ACE simultaneously overcomes the trade-off between low-temperature ion transport and high-temperature interfacial stability. The ACE achieves record-low viscosity (<10 mPa·s) and high ionic conductivity (7.0 mS cm^−1^ at −40 °C), attributed to MA/EC co-solvation behavior revealed by Raman and NMR spectra. Crucially, LiDFP and LiFSI collaboratively construct inorganic-rich SEI/CEI interphases dominated by LiF, Li_3_PO_4_, and Li_2_SO_4_, which enhance interfacial kinetics and suppress degradation. Consequently, LFP|Gr cells deliver zero capacity decay over 500 cycles at 0 °C, 94.1% capacity retention after 100 cycles at 45 °C in Ah-level pouch cells, and stable operation across −40~60 °C with minimal polarization. This tripartite electrolyte design paradigm provides a universal strategy for extreme-condition energy storage systems.

## Figures and Tables

**Table 1:** Ionic conductivities of various electrolytes.

Abbreviation	Formulation	Ionic Conductivity
Baseline	1 M LiPF_6_ in EC:EMC:DMC (*v*:*v*:*v* = 1:1:1)	9.04 mS cm^−1^
MA	1 M LiFSI in MA:EC:EMC (*v*:*v*:*v* = 1:1:1) + 0.5 wt.% LiDFP	16.5 mS cm^−1^
MP	1 M LiFSI in MP:EC:EMC (*v*:*v*:*v* = 1:1:1) + 0.5 wt.% LiDFP	5.73 mS cm^−1^
EA	1 M LiFSI in EA:EC:EMC (*v*:*v*:*v* = 1:1:1) + 0.5 wt.% LiDFP	14.31 mS cm^−1^
EP	1 M LiFSI in EP:EC:EMC (*v*:*v*:*v* = 1:1:1) + 0.5 wt.% LiDFP	10.28 mS cm^−1^
EB	1 M LiFSI in EB:EC:EMC (*v*:*v*:*v* = 1:1:1) + 0.5 wt.% LiDFP	7.69 mS cm^−1^
DFEA	1 M LiFSI in DFEA:EC:EMC (*v*:*v*:*v* = 1:1:1) + 0.5 wt.% LiDFP	7.34 mS cm^−1^
TFEA	1 M LiFSI in TFEA:EC:EMC (*v*:*v*:*v* = 1:1:1) + 0.5 wt.% LiDFP	7.18 mS cm^−1^

**Table 2:** Comparison between our work and previously reported wide-temperature Li batteries.

Reference	Electrolyte	Ionic Conductivity (mS cm^−1^)	Full Cell	Mass Loading (mg cm^−2^)	Room Temperature Discharge Capacity (mAh g^−1^)	Low Temperature Discharge Capacity (mAh g^−1^)	Low Temperature Cycle Performance	High Temperature Discharge Capacity (mAh g^−1^)	High Temperature Cycle Performance
[16]	LiFSI in MP/FEC (9/1)	3.1 (−60 °C)	Gr/LFP (coin cell)	10.5	125 (2C)	78 (−30 °C, 0.1C)	70 (−30 °C, 0.1C, 100th)	130 (60 °C, 0.1C) 115 (80 °C, 0.4C)	/
[14]	LiTFSI in ETFA/FEC (7/3)	0.1 (−70 °C)	Gr/LFP (coin cell)	2.5–4.5	115 (2C)	~90 (−30 °C, 0.1C); ~43 (−60 °C, 0.1C)	/	/	/
[15]	LiFSI in THP	/	Gr/LFP (pouch cell)	/	2.9 Ah (2C)	2.25 Ah (−20 °C, 1C)	/	3 Ah (60 °C, 1C)	/
[13]	LiTFSI in CPME/FEC (7/3)	2.23 (25 °C)	Gr/LFP (pouch cell)	/	0.32 Ah (1C)	0.27 Ah (−20 °C, 1C) 0.2 Ah (−40 °C, 0.1C) 0.1 Ah (−60 °C, 0.1C)	/	/	/
This work	ACE	6.43 (−40 °C)	Gr/LFP (coin/pouch cell)	20	143 (25 °C, 1C) 1.77 Ah (25 °C, 1C)	122 (0 °C, 1C) 108 (−20 °C, 0.1C) 93 (−40 °C, 0.1C) 1.17 Ah (0 °C, 1C) 0.84 Ah (−20 °C, 1C)	112 (0 °C, 0.1C, 500th) 1.25 Ah (0 °C, 0.1C, 100th)	165 (60 °C, 0.1C) 147 (45 °C, 1C) 1.85Ah (45 °C, 1C)	115 (45 °C, 1C, 500th) 1.74 Ah (45 °C, 1C, 100th)

## Data Availability

The original contributions presented in this study are included in the article. Further inquiries can be directed to the corresponding author.
